# Editorial: A year in review: discussions in molecular and structural endocrinology

**DOI:** 10.3389/fendo.2023.1231470

**Published:** 2023-06-12

**Authors:** Alain Couvineau, Ernestina Marianna De Francesco

**Affiliations:** ^1^ Institut National de la Santé et de la Recherche Médicale (INSERM) UMR1149/Inflammation Research Center (CRI), Université Paris Cité, Departement Hospitalo-Universitaire (DHU) UNITY, Faculty of Medicine X, Bichat, Paris, France; ^2^ Endocrinology Unit, Department of Clinical and Experimental Medicine, University of Catania, Garibaldi-Nesima Hospital, Catania, Italy

**Keywords:** endocrinology, obesity, diabetes, cancer, development

A year in review is a research theme on molecular and structural endocrinology from 2021 onwards. Molecular and structural endocrinology focuses on the molecular processes regulating many functions related to endocrine signalling, hormone synthesis/secretion and interactions between hormones, their receptors and interaction with other signaling molecules. This broad spectrum of action involves molecular mechanisms, structural biology, hormonal regulation, genetic, epigenetic and cell signalling, in both physiological and/or pathological contexts such as metabolic diseases encompassing diabetes and obesity, developmental diseases and cancer. Here, contributions by researchers/clinicians offer us a very comprehensive overview of recent findings that extend the current knowledge on several aspects of molecular and structural endocrinology, providing novel challenges and future perspectives in this field.

In an attempt to better clarify how structural changes may affect receptor function and facilitate the establishment of a pathological phenotype, Purba et al. analysed the structure of Epidermal Growth Factor Receptor (EGFR) to determine its ability to auto-activate. In various human cancers, mutations of EGFR promote its aberrant activation, leading to cell proliferation in the absence of ligand. Here, the authors purify full-length EGFR which adopts a homodimeric form in the presence or in the absence of ligand. The ligand was able to stabilize the extracellular domain of the receptor which is very flexible in its absence. This flexibility was associated to the dissociation of the intracellular kinase dimer. Moreover, mutations of residues of the inactive kinase dimer induced auto-activation of EGFR. This structural study of EGFR shows that mutations are able to spontaneously activate the receptor.

Proto-oncogene amplification is a common pathogenetic trait in several cancers. In this regard, Liverani et al. investigated the impact of HRAS proto-oncogene overexpression on lenvatinib treatment in gastroenteropancreatic neuroendocrine tumors (GEP-NET). Lenvatinib, a multi-tyrosine kinase inhibitor, has demonstrated efficacy in GEP-NET patients. In 55% of primary cultures from patients with GEP-NET, lenvatinib showed antitumor activity. This antitumor effect was associated with HRAS overexpression. The Authors show that lenvatinib is highly effective in the treatment of NETs, and that HRAS overexpression could be a reliable marker of its success.

Beyond amplification, genomic alterations such as mutations may strongly contribute to tumorigenesis, therefore their identification in normal vs cancer tissue may pave the way to the identification of better diagnostic and therapeutic tools. In this context, Chorti et al. performed an updated review of the molecular genetics of sporadic parathyroid adenoma, a neoplastic condition affecting 1% of the global population. By analyzing 78 articles, the Authors retrieved several mutated genes and/or proteins differentially expressed or localized in neoplastic vs normal tissue, including CaSR, MEN1, CCND1/PRAD, 41 CDKI, VEGF, FGF, TGFβ and IGF1. Such an effort could have relevant implications in the identification of novel biomarkers for endocrine tumors.

A similar approach has been used for endocrinological diseases other than tumors. For instance, Ahmad et al. analyzed individuals affected by Isolated Growth Hormone Deficiency (IGHD), a disorder associated with reduced circulating levels of GH which causes short stature and skeletal anomalies. In consanguineous family members of patients affected by IGHD, a p.Glu72 mutation in the gene codifying for the growth-hormone-releasing hormone receptor (GHRHR) was identified by whole-exome sequencing (WES) and Sanger sequencing. The pathogenicity of such mutation was further confirmed by performing computational analyses such as structural modeling and docking studies, together with molecular simulation analyses.

Gau et al. studied a novel variant of NR5A1 named R350W in its ability to potentiate NR5A1 functions. NR5A1 is a gene family that encodes transcription factors belonging to the nuclear receptor superfamily. These transcription factors are involved in various biological processes such as gonad development. The authors demonstrated that R350 impaired the transcriptional activities of various genes (hTES, CYP17) which have a strong impact on Sertoli and Leydig cell differentiation. Moreover, R350W could interact with a putative endogenous ligand or co-factor, potentiating NR5A1 activities but not NR5A2. This study demonstrated that orphan factors may play a significant role in human pathophysiology.

Research efforts are directed toward the identification of novel players in obesity and diabetes, due to the growing incidence of these metabolic conditions. In this context, Brustolin et al. studied the impact of TREM-1 (Triggering Receptor of Expressed Myeloid Cells-1) on low-grade inflammation associated with obesity. TREM-1 is involved in many acute inflammation (Septic shock and IBD) and is overexpressed in adipose tissue and liver in obese and diabetic patients. TREM-1 is functionally expressed in subcutaneous and omental adipocytes. A *trem1* KO mouse model associated with a high-fat diet showed limited gain of weight, insulin resistance, and liver inflammation. These results demonstrated that TREM-1 could play a crucial role in obesity development and complications.

Hurley et al. examined the role of ORMDL3 (one of three isoforms of orosomucoid-like proteins), which is identified as a key factor in sphingolipid homeostasis, in pancreatic β-cell functions. A β-cell failure was observed during chronic sphingolipid elevation. The development of a mouse model invalidated for the *ormdl3* gene in pancreatic β-cells showed the absence of alteration in glucose tolerance, insulin sensitivity and secretion, islet morphology, and high fat diet *ormdl3*β^-/-^ mice did not display metabolic alterations. ORMDL3 is not essential for β-cells survival and function on normal and fatty diets. Considering that diabetes may trigger severe clinical issues as observed during pregnancy, Li et al. investigated certain mechanisms through which hyperglycemia leads to obstetrical complications. The authors found that high glucose levels decrease myometrium contractility in patients affected by gestational diabetes mellitus (GDM). Reduced cell contractility was observed also in hypoxic conditions and was paralleled by the up-regulation of HIF-1a (Hypoxia inducible Factor-1a) and TREK1 (TWIK-1-related potassium channel). These findings may provide a mechanistic explanation of the higher rate of obstetrical complications related with GDM.

Other approaches are focused on major diabetes complications; in this regard, Zhao et al. studied the role of growth differentiation factor (GD-10) in wound healing of diabetic foot ulcer (DFU) (a major complication of diabetes mellitus), involving TGF-β1 (Transforming growth factor-beta 1/Smad3 signaling pathway). The use of weighted gene co-expression network analysis (WGCNA) combined with an animal model reproducing DFU, allowed to demonstrate that GD-10 promotes angiogenesis via TGF-β1/Smad3 signaling pathway, leading to a beneficial effect on DFU.

Using high-throughput tools, You et al. mimicked glaucoma *in vitro* by subjecting R28 retinal cells to a pressurized stress model. RNA-seq analyses performed on this model revealed several long-non-coding RNAs (lncRNAs) and microRNAs (mRNAs) differentially regulated and possibly associated with glaucoma. Validation of the cellular data was obtained using clinical samples and confirmed the lncRNAs AC120246.2 and XLOC_006247 as the most strongly increased in glaucoma patients *vs* patients affected by cataracts. These findings set the stage for broadening the diagnostic and therapeutic perspectives of glaucoma patients.

The cross-talk between hormone and growth-factor receptors is known to play a key regulatory role in signaling transduction, potentially affecting endocrine homeostasis in normal and pathological conditions. Mistry et al. demonstrated that Fibroblast Growth 21 (FGF21) is directly implicated in the resistance to Growth Hormone (GH) and in the failure of linear growth in very pre-term (VPT) infants. By using rare and unique human growth plates obtained from children, the Authors found that FGF21 decreases GHR half-life and inhibits downstream GHR signaling events (i.e. IGF-1 release). In VPT infants, FGF21 levels were higher in the stage of deflected linear growth, whereas in the phase of catch-up growth a reduction of FGF21 levels was observed, together with an increase of IGF1 levels. These findings suggest that FGF21 may dampen GH/GHR signaling toward growth failure.

## Author contributions

All authors listed have made a substantial, direct and intellectual contribution to the work, and approved it for publication.

